# Correction to: LncRNA AK023391 promotes tumorigenesis and invasion of gastric cancer through activation of the PI3K/Akt signaling pathway

**DOI:** 10.1186/s13046-020-01656-1

**Published:** 2020-08-12

**Authors:** Yanxia Huang, Jing Zhang, Lidan Hou, Ge Wang, Hui Liu, Rui Zhang, Xiaoyu Chen, Jinshui Zhu

**Affiliations:** 1 Department of Gastroenterology, Shanghai Jiao Tong University Affiliated Sixth People’s Hospital, No. 600 Yishan Road, Shanghai, 200233 China; 2 Department of Gastroenterology, Shanghai Ninth People’s Hospital, Shanghai Jiao Tong University School of Medicine, Shanghai, China

**Correction to: J Exp Clin Cancer Res 36, 194 (2017)**

**https://doi.org/10.1186/s13046-017-0666-2**

Following publication of the original article [1], the authors identified an error in Fig. 5; specifically the HGC-27 cell line in figure 5A. The correct figure is given below.

**Fig. 5:**
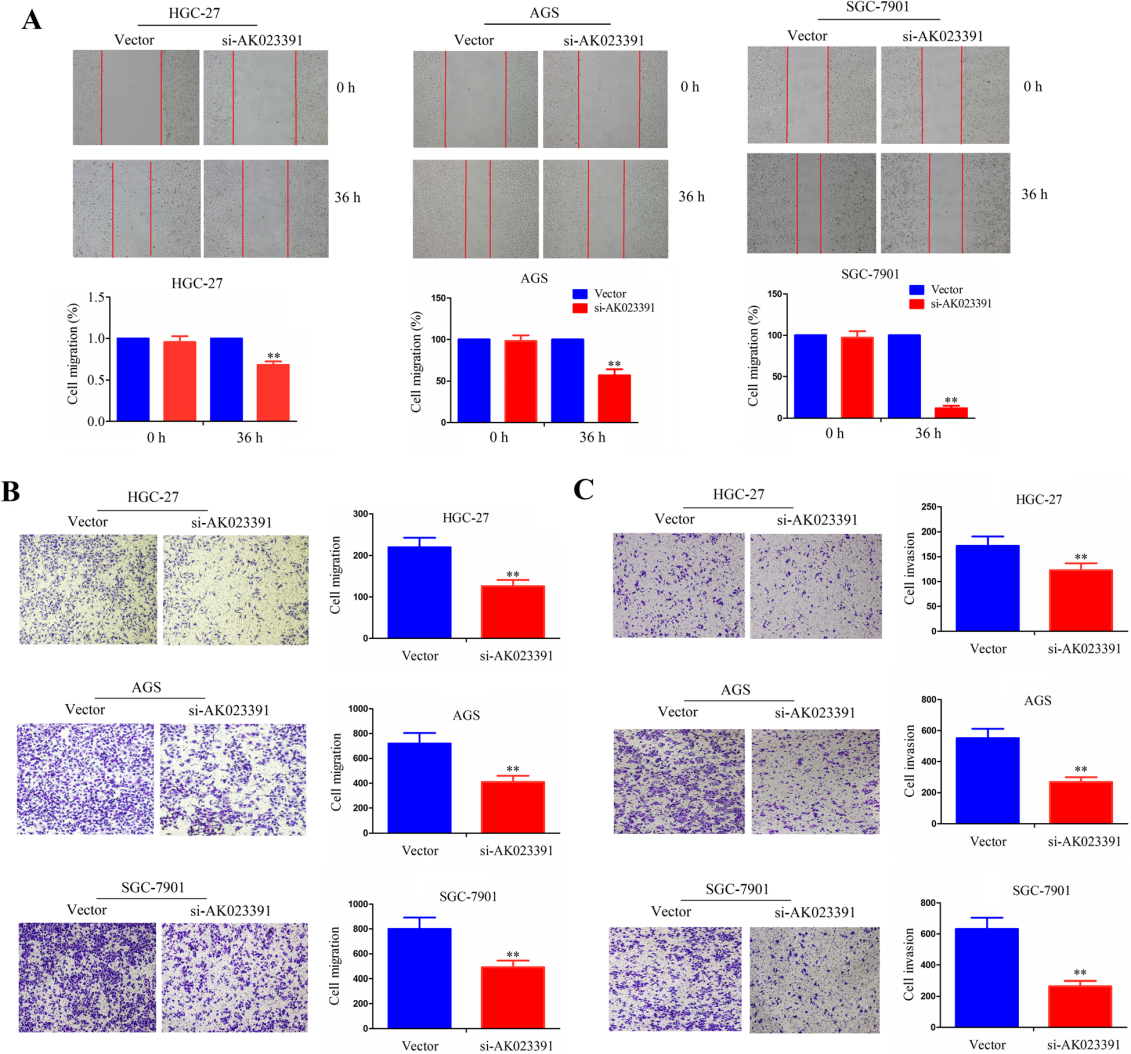
Knockdown of AK023391 inhibited migration and invasion of gastric cancer (GC) cells. **a**-**b** Cell migration abilities were respectively determined by the wound-healing assay and Transwell migration assay in si-AK023391-transfected HGC 27, AGS, and SGC-7901 cells. **c** The cell invasive potential was assessed by the Transwell invasion assay in si-AK023391-transfected HGC 27, AGS, and SGC-7901 cells. **P < 0.01
